# Lead-Related Genetic Loci, Cumulative Lead Exposure and Incident Coronary Heart Disease: The Normative Aging Study

**DOI:** 10.1371/journal.pone.0161472

**Published:** 2016-09-01

**Authors:** Ning Ding, Xin Wang, Marc G. Weisskopf, David Sparrow, Joel Schwartz, Howard Hu, Sung Kyun Park

**Affiliations:** 1 Department of Epidemiology, School of Public Health, University of Michigan, Ann Arbor, Michigan, United States of America; 2 Department of Environmental Health, Harvard T.H. Chan School of Public Health, Boston, Massachusetts, United States of America; 3 Veterans Affairs Normative Aging Study, Veterans Affairs Boston Healthcare System, Boston, Massachusetts, United States of America; 4 Department of Medicine, Boston University School of Medicine, Boston, Massachusetts, United States of America; 5 Dalla Lana School of Public Health, University of Toronto, Toronto, Ontario, Canada; 6 Department of Environmental Health Sciences, School of Public Health, University of Michigan, Ann Arbor, Michigan, United States of America; Stony Brook University, Graduate Program in Public Health, UNITED STATES

## Abstract

**Background:**

Cumulative exposure to lead is associated with cardiovascular outcomes. Polymorphisms in the δ-aminolevulinic acid dehydratase (*ALAD*), hemochromatosis (*HFE*), heme oxygenase-1 (*HMOX1*), vitamin D receptor (*VDR*), glutathione S-transferase (*GST*) supergene family (*GSTP1*, *GSTT1*, *GSTM1*), apolipoprotein E (*APOE*),angiotensin II receptor-1 (*AGTR1*) and angiotensinogen (*AGT*) genes, are believed to alter toxicokinetics and/or toxicodynamics of lead.

**Objectives:**

We assessed possible effect modification by genetic polymorphisms in *ALAD*, *HFE*, *HMOX1*, *VDR*, *GSTP1*, *GSTT1*, *GSTM1*, *APOE*, *AGTR1* and *AGT* individually and as the genetic risk score (GRS) on the association between cumulative lead exposure and incident coronary heart disease (CHD) events.

**Methods:**

We used K-shell-X-ray fluorescence to measure bone lead levels. GRS was calculated on the basis of 22 lead-related loci. We constructed Cox proportional hazard models to compute adjusted hazard ratios (HRs) and 95% confidence intervals (CIs) for incident CHD. We applied inverse probability weighting to account for potential selection bias due to recruitment into the bone lead sub-study.

**Results:**

Significant effect modification was found by *VDR*, *HMOX1*, *GSTP1*, *APOE*, and *AGT* genetic polymorphisms when evaluated individually. Further, the bone lead-CHD associations became larger as GRS increases. After adjusting for potential confounders, a HR of CHD was 2.27 (95%CI: 1.50–3.42) with 2-fold increase in patella lead levels, among participants in the top tertile of GRS. We also detected an increasing trend in HRs across tertiles of GRS (p-trend = 0.0063).

**Conclusions:**

Our findings suggest that lead-related loci as a whole may play an important role in susceptibility to lead-related CHD risk. These findings need to be validated in a separate cohort containing bone lead, lead-related genetic loci and incident CHD data.

## Introduction

Incident coronary heart disease (CHD) is a major health problem, and the leading cause of mortality worldwide [1]. Environmental toxicants, such as lead (Pb) and other heavy metals, are known to be associated with cardiovascular disease [2]. Weisskopf et al. reported an increased risk for future ischemic heart disease (IHD, also known as CHD) mortality with higher blood and bone lead levels in the VA Normative Aging Study (NAS) [3]. Several mechanisms could potentially explain lead’s association with cardiovascular outcomes, including reduction in renal function, induction of oxidative stress and inflammation, stimulation of the renin-angiotensin system, as well as endothelial dysfunction [4–6].

Blood lead levels have been profoundly reduced in the U.S population over the last 30 years [6]. Although health-based guidelines limiting environmental and occupational exposures to lead have become more stringent to protect the population against adverse health effects, long-term lead exposure is still responsible for potential health risks [7,8]. In addition, genetically susceptible individuals may not be fully protected by current regulatory standards. It has been increasingly clear that individual genetic backgrounds influence predisposition to lead toxicity [2]. These genes can be divided into two groups: genes that influence lead uptake and retention (known as toxicokinetics), and genes that alter toxic effects of lead (known as toxicodynamics). A number of genes and gene encoding proteins have been identified to play important roles in lead toxicokinetics and/or toxicodynamics: δ-aminolevulinic acid dehydratase (ALAD), an enzyme on the heme-biosynthetic pathway that binds over 80% of lead in erythrocytes [9]; hemochromatosis (HFE), a membrane protein that regulates uptake of cellular iron and other divalent metals including lead [10]; heme oxygenase-1 (HMOX1), a heme-degrading enzyme that plays an important role in the inflammation and oxidative stress induced by lead [11–14]; vitamin D receptor (VDR) that plays a role in calcium homeostasis that influences the absorption and retention of lead into blood and bone [15]; apolipoprotein E (APOE) that affects lipid metabolism, down-regulates blood lead concentrations, and possesses antioxidative property [16–18]; glutathione S-transferases (GSTs), a family of phase-II detoxification isozymes involved in catalyzing the conjugation of lead and glutathione to form a thermodynamically stable complex, which decreases lead bioavailability and protects against lead toxicity through reduced oxidative stress [19–22]; and the renin-angiotensin system involved in the development of hypertension where lead exposure may enhance blood angiotensin I levels by increasing plasma renin activity, which in turn contribute to production and activation of substrate-induced angiotensin converting enzyme [23,24]. Epidemiologic studies have identified that these genes may modify the association between lead exposure and cardiovascular and other health outcomes [13,18,25–27]. However, these previous studies focused on one or a few of the lead-related genetic polymorphisms. No study integrating multiple genetic polymorphisms in lead toxicokinetics and toxicodynamics has been conducted.

The purpose of this study was to evaluate effect modification of the association between cumulative exposure to lead and incident CHD events by genes related to lead toxicokinetics and toxicodynamics. We hypothesized that in the Normative Aging Study (NAS), the lead-CHD association would be stronger among participants with higher allelic risk profiles. The genes under consideration in this analysis are the *ALAD* gene, *HFE* gene, *HMOX1* gene, *VDR* gene, *GST* supergene family (*GSTT1* and *GSTM1*), *APOE* gene, angiotensin II receptor-1 (*AGTR1*) gene, and angiotensinogen (*AGT*) gene. In this study, we tested whether not only single nucleotide polymorphisms (SNPs) in each of those lead-related genes, but also integrated measures of all those polymorphisms and genetic risk score (GRS), modified the association between bone lead and incident CHD.

## Materials and Methods

### Ethics statement

All participants provided written informed consent. This study was reviewed and approved by the Institutional Review Boards of each participating institute, the University of Michigan School of Public Health, the Harvard School of Public Health and the Department of Veterans Affairs Boston Healthcare System.

### Study population

The NAS is a longitudinal study of aging initiated by the Veterans Administration in Boston, Massachusetts in 1963. Participants were 2280 healthy males, mostly white, ranging from 21 to 80 years of age. All subjects enrolled in the study were free of past or present chronic medical conditions, including heart disease, cancer, diabetes, peptic ulcer, gout, bronchitis, sinusitis, recurrent asthma, or hypertension. Each participant returned for examination every 3 to 5 years.

Starting from August 1991, NAS participants who gave informed consent were invited to undergo K-shell-X-ray fluorescence (KXFR) bone lead measurements at the Ambulatory Clinical Research Center of the Brigham and Women’s Hospital in Boston, Massachusetts. Of the 1283 participants who actively attended regular NAS examinations between August 1^st^ 1991 and August 8^th^ 2002, 878 participants had their bone lead measured. After excluding participants with high bone lead uncertainty (3 for patella and 6 for tibia lead), participants with negative lead levels (3 for patella and 6 for tibia lead), participants with missing values of the potential confounding factors (29 for patella and 30 for tibia lead), as well as participants with missing values of variables used in the prediction of enrollment into the bone lead study (8 for both patella and tibia lead, see more details of constructing inverse probability weighting in Statistical Analysis), 604 participants for patella lead and 607 participants for tibia lead, without a history of CHD (angina pectoris, myocardial infarction or CHD death) at the time of bone lead measurement visit, were included. The final analyses considered 589 participants (583 for patella lead, and 588 for tibia lead), with at least one SNP in *ALAD*, *HFE*, *HMOX1*, *VDR*, *APOE*, *GSTP1*, *GSTT1*, *GSTM1*, *AGTR1* and *AGT*.

### Case ascertainment

Coronary heart disease (CHD) cases were defined as myocardial infarction, angina pectoris, or CHD deaths. We asked participants their history of heart disease since last visit. Board-certified cardiologists review each report of CHD events. Myocardial infarction was defined according to pathologic Q wave changes, increases in serum glutamic-oxaloacetic transaminase and lactic dehydrogenase levels, as well as concurrent chest discomfort consistent with myocardial infarction, or by autopsy. Angina pectoris was defined by recurrent chest discomfort lasting up to 15 min and related to exertion and relieved by rest or nitroglycerin. CHD deaths were ascertained from death certificates based on the 9^th^ revision of the International Classification of Disease (ICD-9) codes 410 to 414 and 429.2.

### Bone lead measurement

NAS participants had their bone lead levels measured at both the patella and mid-tibial shaft by using ABIOMED K-shell-X-ray instrument, as described in detail previously [28]. The measurement of bone lead, with unit of μg/g bone mineral, consists of an unbiased estimate and an estimate of uncertainty. 3 and 6 participants with uncertainty for patella or tibia lead, higher than 10 or 15 μg/g, respectively, were excluded since high levels of uncertainties likely represented less than optimal measurements due to excessive motion during the measurement, or a degraded signal of X-ray [29]. Both patella and tibia lead measurements were used as indices of cumulative lead levels in humans. Patella is predominantly made up of trabecular-type bone; while, for tibia, the major component is cortical-type bone. Lead in the patella is more mobilizable than that in the tibia, representing a half-life of decades for tibia lead and a half-life of a few years for lead in the patella [30].

### Selection of gene polymorphisms

Candidate gene selection was based on functionality, which had been found in biological and epidemiological studies evaluating associations between lead and genetic variants. The next step was to identify ‘functional’ polymorphisms (i.e. non-synonymous and splice junction SNPs), synonymous and noncoding SNPs. Presently known polymorphisms in the lead-related genes are shown in Table 1.

**Table 1:** Gene polymorphisms affecting lead toxico-kinetics and toxico-dynamics.

Gene	SNP	Minor Frequency Allele	SNP Location	Reported Functions	References
**δ-aminolevulinic acid dehydratase gene (*ALAD*)**	rs1800435	C	Exon 4^a^	The presence of *ALAD2* allele was associated with higher blood lead levels in some occupational cohorts with high exposure, whereas others have not. Blood lead levels at background exposure levels show no clear association with *ALAD* genotype, although a small study showed *ALAD2* was associated with lower blood lead levels in Japanese population.	Wetmur et al., 1994 [31]; Alexander et al., 1998 [32]; Schwartz et al., 1995 [33]; Hu et al., 2001 [34]; Pawlas et al., 2012 [35]; Miyaki et al., 2009 [36];
**Vitamin D receptor (*VDR*)**	rs1544410	A	Intron 8	*Bsm1* (rs1544410) and *Fok1* (rs10735810) affects bone mineral density and both blood/tibia lead levels; *Apa1* (rs7975232) affects serum lead levels for the haplotype combined with *Bsm1* and *Fok1*; Significant effect modification by *Bsm1* or *Taq1* (rs731236) found in the association between bone lead and pulse pressure.	Hurwitz et al., 1996 [37]; Haynes et al., 2003 [38]; Cooper et al., 1996 [39]; Rezende et al., 2007 [40]; Jhun et al., 2015 [25];
	rs731236	C	Exon 9^a^		
	rs7975232	C	Intron 8		
	rs1073581	A	Exon 2^a^		
	rs757343	A	Intron 8		
**Hemochromatosis gene (*HFE*)**	rs1799945	G	Exon 2^a^	C282Y (rs1799945) and H63D (rs1800562) are associated with hemochromatosis, affecting iron uptake; They are also found to influence bone lead absorption and oxidative stress.	Waheed et al., 1997 [41]; Wright et al., 2004 [42];
	rs1800562	A	Exon 4^a^		
**Heme oxygenase-1 gene (*HMOX1*)**	rs2071746	T	Promoter	rs2071746, rs2071747 and rs2071749 are associated with regulation of expression of antioxidant enzymes HMOX1; Long (GT)n repeats in the *HMOX1* promoter may reduce heme oxygenase inducibility by reactive oxygen species.	Tanaka et al., 2011 [14]; Ashrafunnisa et al., 2014 [11]; Park et al., 2009 [13];
	rs2071747	C	Exon 1^a^		
	rs2071749	A	Intron 3		
	rs5995098	G	Intron 4		
	(GT)n repeats	Long^b^	Promoter		
**Apolipoprotein E gene (*APOE*)**	rs440446	C	Intron 1	*APOE ε4* carriers would have depressed nitric oxide bioavailability, and contribute to lead-related hypertension; *APOE ε4* also possesses reduced antioxidant abilities, bound metal ions, and magnified their cytotoxicity; rs405509 and rs440446 could be associated with cortical bone strength, which is associated with tibia lead levels; rs769446 is associated with the plasma APOE concentration.	Colton et al., 2002 [43]; Miyata et al., 1996 [44]; Tolonen et al., 2011 [45]; Theppeang et al., 2008 [46]; Mannila et al., 2013 [47];
	rs405509	C	Promoter		
	rs449647	A	Promoter		
	rs7412	T	Exon 4^a^		
	rs429358	C	Exon 4^a^		
	rs769446	C	Promoter		
**Glutathione S- transferase pi 1 gene (*GSTP1*)**	rs1695	G	Exon 5^a^	*GSTM1*, *GSTP1* and *GSTT1* may alter the responses to oxidative stress and inflammatory process caused by lead exposure; *GSTT1* deletion, *GSTM1* deletion, or at least one risk allele of *GSTP1* rs1695 are associated with strengthened oxidative stress and inflammation.	Ercal et al., 2001 [48]; Sirivarasai et al., 2013 [22]; Coral-Vazquez et al., 2013 [49];
**Glutathione S- transferase theta 1 gene (*GSTT1*)**^c^	--	Gene Deletion	--		
**Glutathione S- transferase mu 1 gene (*GSTM1*)** ^c^	--	Gene Deletion	--		
**Angiotensinogen gene (*AGT*)**	rs699	C	Exon 2^a^	*AGT* M268T (rs699), rs5046, rs5050 and rs2493137 are associated with lead-induced hypertension; *AGTR1* is associated with hypertension.	Kim et al., 2014 [26]; Warrell et al., 2010 [50]; Li et al., 2014 [51]; Nie et al., 2009 [52];
	rs5046	T	Promoter		
	rs5050	G	Promoter		
	rs2493137	C	Promoter		
**Angiotensin II receptor-1 gene (*AGTR1*)**	rs12695908	G	Intron 3		

^a^ Amino acid substitution in exons: rs1800435 Lys59Asn, rs731236 Ile352Ile, rs1799945 His63Asp, rs1800562 Cys282Try, rs2071747 Asp7His, rs7412 Arg176Cys, rs429358 Cys130Arg, rs1695 Ile105Val, and rs699 Met268Thr.

^b^ The numbers of (GT)n repeats were classified as class Short (<27 (GT)n repeats), class Medium (27–32 (GT)n repeats), and class Large (≥33 (GT)n repeats).

^c^ *GSTT1* and *GSTM1* polymorphisms are considered as deletion vs. no-deletion in our study.

### Genotyping

Multiplex polymerase chain reaction (PCR) assays were designed with Sequenom SpectroDESIGNER software (Sequenon, Inc, San Diego, CA) by inputting sequences containing the SNP site and 100 base pairs of flanking sequence on either side of the SNP. A total of 24 SNPs were included: *ALAD* rs1800435; *VDR* rs1544410, rs731236, rs7975232, rs1073581, rs757343; *HFE* rs1799945, rs1800562; *HMOX1* rs2071746, rs2071747, rs2071749, rs5995098; *APOE* rs440446, rs405509, rs449647, rs7412, rs429358, rs769446; *GSTP1* rs1695; *AGTR1* rs12695908; and *AGT* rs699, rs5046, rs5050, rs2493137. *GSTM1* and *GSTT1* were deletion polymorphisms. The assay consists of PCR amplification of exons 4 and 5 of the *GSTM1* allele and concomitant amplification of the cytochrome P450 1A1 (*CYP1A1*) gene as a positive control. PCR products indicate the presence of one or more copies of the gene. The *HMOX1* gene was also genotyped for length polymorphism. Microsatellite (GT)n-length assay was designed as described by Yamada et al. [53]. PCR products at the 5’-flanking region of *HMOX1* were analyzed with a laser-based automated DNA sequencer, and the numbers of (GT)n repeats were classified according to the previous study [13], as class S (<27 (GT)n repeats), class M (27–32 (GT)n repeats), and class L (≥33 (GT)n repeats). The pass rate of genotyping for these SNPs varied between 94% and 98.6%.

### Genetic risk scores

We selected polymorphisms with plausible biological relationships to the pathophysiology of lead-induced CHD. The genetic risk score 1 was constructed by summing up all 22 available SNPs. The genetic risk score 2 was constructed by using 9 SNPs which we found significantly modified the relationship between bone lead and incident CHD events in our study. The 9 SNPs were *VDR* (rs1544410, rs7975232, rs757343), *HMOX1* (rs2071746, rs2071749, rs5995098), *APOE* (rs429358), and *AGT* (rs699, rs5046).

### Covariates

Data on all covariates were obtained from regular NAS visits since the time of the baseline bone lead measurements. Body mass index (BMI) was calculated based on height (cm) and weight (kg) assessment. We considered cigarette smoking as both status indicators (never/ever) and pack-years. We categorized education as less than high school, high school, some college and no less than 4 years graduate study. Blood samples were analyzed for serum high-density lipoprotein (HDL) and total cholesterol levels. Hypertension were defined as systolic blood pressure greater than 140 mmHg and diastolic blood pressure greater than 90 mmHg, any medications before, or ever diagnosed with hypertension by a physician. Diabetes were defined as fasting glucose levels greater than 126 mg/dL, or ever diagnosed with diabetes by a physician.

### Statistical analysis

Univariate statistics were calculated and examined for cases and non-cases of CHD. We used Chi-square or exact Fisher tests for categorical variables, and t test for continuous variables. For SNPs, we calculated allele frequencies and assessed Hardy-Weinberg equilibrium for each genotype.

We computed person time for each individual from baseline to the date of first CHD event (angina pectoris or myocardial infarction) or CHD death, whichever came first; and for non-cases, from the date of bone lead measurement to the last active follow-up visit until June, 2011. We used Cox proportional hazard regression models to calculate hazard ratios (HRs) and 95% confidence intervals (CIs) for the development of new CHD in relation to lead exposure. Bone lead variables were log_2_-transformed, modeled as linear continuous variables. HRs and 95% CIs were, therefore, presented for a two-fold increase in bone lead levels to facilitate interpretation of the effect estimates.

To assess effect modification by different genes, we included log-transformed lead levels, indicators for genotype classification, and cross product terms between lead biomarker and genotype. We also controlled for age, smoking status, BMI and the ratio of total cholesterol to HDL cholesterol level. Hypertension is a well-known risk factor for CHD. However, sufficient evidence revealed a causal relationship between lead exposure and hypertension. We, therefore, decided not to include hypertension or blood pressure in our models because hypertension could be in the causal pathway (i.e. overadjustment bias) [54]. In addition, we constructed an unweighted multi-locus GRS (GRS 1) for each individual by simply summing up the number of risk alleles (0/1/2) for all 22 available SNPs. We found *VDR* rs1544410 and rs731236 are in high linkage disequilibrium (r^2^ = 0.97), also similar for *VDR* rs7975343 and rs1073581 (r^2^ = 0.99) (linkage disequilibrium results are shown in Tables A-D in S1 File). For this reason we exclude rs731236 and rs1073581 in our GRS. We also created another GRS (GRS 2) using 9 and 8 SNPs that significantly modified the patella lead-CHD and tibia lead-CHD associations, respectively. We computed false discovery rates (FDRs) from p-values using the Benjamini-Hochberg method to adjust for multiple comparisons [55].

In our study population, collider stratification bias might exist and produce misleading results, since selection into the KXRF bone lead sub-study was linked to both cardiovascular diseases and potential confounders at the time of enrollment [3]. To mitigate the bias, we assigned weights to the subjects based on inverse probability weighting (IPW) as per Weisskopf et al. [3]. Briefly, a single logistic regression model was constructed to predict the probability of sub-study entrance, with one record per study visit at the time of bone lead measurements for those participants in the sub-study; for those without bone lead measured, we utilized the last visit before 2002 (the last year of the bone lead measurements used in our study). The C statistic from this model was 0.87. All the predictors incorporated in the logistic regression model are presented in S1 Table.

We conducted sensitivity analyses to verify analytical consistency. We investigated possible intermediates by incorporating blood pressure into the models. We replaced ever/never smoking indicator with smoking in pack-years in our models. We also tried to mitigate the potential selection bias by restricting our study population to those who were 45 years or younger at the entry into the NAS study, combined with IPW [3]. All the above analyses were performed using SAS, version 9.4 (SAS Institute, Inc., Cary, North Carolina) and R version 3.2.2 for the multiple comparison correction (R Core Team 2014).

### Power Calculations

We calculated post-hoc powers for the gene-environment interaction terms using Quanto 1.2.4, which is a web-based software program to compute either sample size or power in studies of gene-gene or gene-environment interaction (http://biostats.usc.edu/Quanto.html). Since the Quanto software can only allow five types of study designs (i.e., “unmatched case-control”, “matched case-control”, “case-sibling”, “case-parent”, and “case-only”), we assumed that our study design was a standard unmatched case-control (not a prospective cohort) consisting of a random sample of unrelated subjects. Under this setting, we assumed our observed HRs to approximate odds ratios. In addition, we also assumed the dominant inheritance model (i.e. risk allele carriers vs. participants with no risk allele) and a two-sided test with type-1 error of 0.05.

## Results

S2 Table shows that genotype frequencies, and incident CHD events by *ALAD*, *HFE*, *HMOX1*, *VDR*, *GSTP1*, *GSTT1*, *GSTM1*, *APOE*, *AGTR1*, and *AGT* genotypes. All the 24 SNPs were in Hardy-Weinberg equilibrium with p-value>0.10, except for *HFE* rs1799945 (p = 0.03), and *AGT* rs5046 (p = 0.003) (S3 Table). Data are not shown in the table for length polymorphisms and deletion polymorphisms. 72 (13.3%) subjects were *HMOX1* L-allele carriers; 75 (19.4%) subjects were with *GSTT1* deleted; 283 (54.5%) subjects were with *GSTM1* deleted. CHD cases and non-cases did not differ by minor allele profiles, except for *APOE* rs449647 (χ^2^ = 8.97, p-value = 0.01). There was no significant difference in bone lead levels by genotypes (S4 Table).

Of 589 subjects in our study, 136 subjects had been diagnosed as CHD cases during the follow-up periods (Table 2). Participants were followed for up to 20 years. The mean age at the time of bone lead measurement among the study population was 66 years (range: 48–96 years). Although cholesterol and BMI were significantly higher, the distribution of other covariates, including known risk factors for CHD such as smoking, socioeconomic status (i.e. education), systolic and diastolic blood pressure, and diabetes status were not significantly different between cases and non-cases.

**Table 2:** Baseline Characteristics of CHD cases and non-cases, Normative Aging Study (n = 589).

	No CHD (n = 453)		CHD (n = 136)		
**Main Exposure**					
	**Total**^c^	**Mean ± SD**	**Total**	**Mean ± SD**	**P value**^a^
**Patella lead**	449	29.2 ± 18.1	135	32.1 ± 18.8	0.12
**Tibia lead**	452	20.2 ± 12.5	136	22.6 ± 13.5	0.03
**Continuous variables**					
**Age (years)**	453	66.1 ± 7.4	136	65.8 ± 6.6	0.63
**Follow-up time (years)**	453	9.7 ± 5.8	136	9.0 ± 5.1	0.10
**Body mass index (kg/meter**^**2**^**)**	453	27.5 ± 3.5	136	28.6 ± 3.9	<0.01
**Smoking (pack-years)**	444	21.0 ± 25.6	133	22.3 ± 25.3	0.35
**Total cholesterol (mg/dL)**	453	226.8 ± 35.6	136	234.2 ± 39.5	0.03
**High-density lipoprotein (mg/dL)**	453	49.2 ± 13.3	136	47.8 ± 11.1	0.21
**Total cholesterol/HDL ratio**	453	4.9 ± 1.4	136	5.1 ± 1.2	0.13
**Categorical variables**					
	**Total**^c^	**N (%)**	**Total**	**N (%)**	**P value**^b^
**Smoking**	453		136		0.19
**Never smokers**		147 (32.5)		36 (26.5)	
**Ever smokers**		306 (67.5)		100 (73.5)	
**Education**	433		131		0.60
**<high school**		42 (9.7)		18 (13.7)	
**high school**		149 (34.4)		45 (34.4)	
**some college**		110 (25.4)		32 (24.4)	
**≥ 4 year college graduate**		132 (30.5)		36 (27.5)	
**Hypertension**	453	201 (44.4)	136	92 (47.8)	0.48
**Diabetes**	453	51 (11.3)	136	17 (12.5)	0.69

^a^ P value from t-test or Wilcoxon rank-sum test (patella and tibia lead, total cholesterol, and total cholesterol to HDL ratio).

^b^ P value from Chi-square test.

^c^ Totals vary because of missing data.

After incorporating interactions between bone lead and genotypes into the models, we found that 5 and 4 polymorphisms reached statistical significance (p<0.05) and borderline significance (p<0.1), respectively, when genotypes were modeled as additive (Table 3). Using the dominant genetic model, 4 and 3 polymorphisms reached statistical significance and borderline significance (S5 Table).

**Table 3:** Adjusted estimates^a^ of CHD per 2-fold increase in patella lead levels, stratified by different gene polymorphisms.

Associations in the entire population					
			95%Confidence Interval		
	N	Hazard Ratio^b^	Lower	Upper	P value^c^
**Crude model**	583	1.42	1.20	1.68	< .0001
**Adjusted model**	583	1.36	1.15	1.61	0.0004
**Associations by genotype of each lead-related SNPs**					
***Vitamin D (1*,*25-dihydroxyvitamin D3) receptor gene (VDR gene)***					
***VDR rs1544410 (Bsm1)***	494				0.004
**G/G**		0.97	0.73	1.28	
**G/A**		1.59	1.20	2.10	
**A/A**		1.77	1.18	2.68	
***VDR rs731236 (Taq1)***	521				0.009
**T/T**		1.00	0.76	1.33	
**T/C**		1.56	1.19	2.04	
**C/C**		1.70	1.15	2.52	
***VDR rs7975232 (Apa1)***	520				0.007
**A/A**		1.48	1.11	1.97	
**A/C**		1.71	1.30	2.25	
**C/C**		0.84	0.62	1.13	
***VDR rs1073581 (Fok1)***	509				0.09
**G/G**		1.19	0.91	1.56	
**G/A**		1.28	1.01	1.63	
**A/A**		2.11	1.19	3.74	
***VDR rs757343 (Tru91)***	516				0.07
**G/G**		1.17	0.89	1.52	
**G/A**		1.30	1.02	1.64	
**A/A**		2.09	1.18	3.73	
***δ-aminolevulinic acid dehydratase gene (ALAD gene)***					
***ALAD rs1833435***	545				0.84
**A/A**		1.34	1.20	1.60	
**A/T**		1.36	0.74	2.50	
**T/T**		1.19	0.24	5.93	
***Hemochromatosis gene (HFE gene)***					
***HFE rs1799945 (H63D)***	509				0.25
**C/C**		1.41	1.15	1.72	
**G/C**		1.10	0.79	1.56	
**G/G**		1.67	0.26	10.78	
***HFE rs1800562 (C282Y)***	510				0.22
**G/G**		1.36	1.13	1.64	
**G/A**		0.99	0.58	1.69	
**A/A**		--^d^	--	--	
***Heme oxygenase 1 gene (HMOX1 gene)***					
***HMOX1 rs2071746***	516				0.09
**A/A**		1.51	1.07	2.13	
**A/T**		1.44	1.13	1.83	
**T/T**		1.02	0.74	1.41	
***HMOX1 rs2071749***	514				0.10
**G/G**		1.02	0.77	1.35	
**G/A**		1.54	1.21	1.98	
**A/A**		1.34	0.91	1.99	
***HMOX1 rs5995098***	519				0.15
**C/C**		1.62	1.23	2.14	
**C/G**		1.18	0.94	1.49	
**G/G**		1.40	0.78	2.51	
***HMOX1 rs2071747***	510				0.82
**G/G**		1.36	1.13	1.63	
**G/C**		1.45	0.81	2.61	
**C/C**		--	--	--	
***HMOX1 length polymorphisms***	541				0.18
**S or M alleles**		1.46	1.20	1.77	
**Any L allele**		1.09	0.74	1.60	
***Alipoprotein E gene (APOE gene)***					
***APOE rs429358***	500				0.10
**T/T**		1.43	1.17	1.75	
**T/C**		1.05	0.75	1.48	
**C/C**		0.87	0.39	1.95	
***APOE rs440446***	521				0.64
**G/G**		1.25	0.95	1.63	
**G/C**		1.42	1.09	1.86	
**C/C**		1.34	0.83	2.16	
***APOE rs405509***	534				0.33
**A/A**		1.22	0.86	1.74	
**A/C**		1.26	0.99	1.60	
**C/C**		1.58	1.10	2.28	
***APOE rs449647***	513				0.60
**T/T**		1.30	1.05	1.61	
**T/A**		1.60	1.15	2.21	
**A/A**		1.19	0.45	3.16	
***APOE rs7412***	540				0.30
**C/C**		1.34	1.10	1.64	
**C/T**		1.45	0.98	2.16	
**T/T**		2.42	0.57	10.25	
***APOE rs769446***	509				0.46
**T/T**		1.32	1.08	1.61	
**T/C**		1.87	1.20	2.91	
**C/C**		0.47	0.11	2.05	
***Angiotensinogen gene (AGT gene)***					
***AGT rs699***	485				0.02
**T/T**		2.17	1.51	3.12	
**T/C**		1.29	1.01	1.64	
**C/C**		1.18	0.82	1.7	
***AGT rs5046***	487				0.05
**C/C**		1.57	1.27	1.94	
**C/T**		1.13	0.83	1.53	
**T/T**		1.04	0.35	3.09	
***AGT rs5050***	483				0.75
**T/T**		1.36	1.09	1.69	
**T/G**		1.39	1.02	1.91	
**G/G**		--	--	--	
***AGT 2493137***	485				0.25
**T/T**		1.61	1.20	2.15	
**T/C**		1.37	1.08	1.74	
**C/C**		1.07	0.53	2.18	
***Angiotensin II receptor 1 gene (AGTR1 gene)***					
***AGTR1 rs12695908***	486				0.72
**C/C**		1.44	1.20	1.74	
**C/T**		1.25	0.63	2.50	
**T/T**		--	--	--	
***Glutathione S-transferase pi 1 gene (GSTP1 gene)***					
***GSTP1 rs1695***	496				0.31
**A/A**		1.39	1.10	1.77	
**A/G**		1.23	0.93	1.62	
**G/G**		0.78	0.31	2.00	
***Glutathione S-Transferase Theta 1 (GSTT1 gene)***					
***GSTT1***	386				0.16
**Deletion**		1.21	0.84	1.76	
**No deletion**		1.65	1.32	2.08	
***Glutathione S-Transferase Mu 1 (GSTM1 gene)***					
***GSTM1***	519				0.15
**Deletion**		1.56	1.21	2.01	
**No deletion**		1.21	0.95	1.54	

^a^ Hazard ratio and 95% confidence interval with adjustment for age, BMI, smoking status (ever/never) and total cholesterol to HDL cholesterol ratio for all models.

^b^ Hazard ratio indicates HR of CHD events for 2-fold increase in lead levels.

^c^ P value from Wald test in the adjusted Cox proportional hazard models, when genotype indicators treated as a continuous variable.

^d^ Null values are due to the absence of subjects or a small number of subjects.

For *VDR* rs1544410 (*Bsm1*), rs731236 (*Taq1*), rs757343 (*Fok1*), rs757343 (*Tru91*) and *HMOX1* rs2071749, a 2-fold increase in patella lead was associated with HRs of incident CHD by 1.65 (95%CI: 1.31–2.08), 1.61 (95%CI: 1.29–2.02), 1.47 (95%CI: 1.17–1.83), 1.48 (95%CI: 1.18–1.85), and 1.51 (95%CI: 1.22–1.86), respectively, among subjects with at least one minor allele; whereas no significant associations were found among those with homozygous major alleles. For VDR rs7975232 (*Apa1*), a 2-fold increase in patella lead was associated with a HR of CHD by 1.48 (95%CI: 1.11–1.97) among subjects with no minor allele, and a HR of CHD by 1.71 (95%CI: 1.30–2.25) among subjects with only one minor allele; however, no significant association was found among those with two minor alleles. On the contrary, among subjects without any minor allele in *HMOX1* rs2071746, a 2-fold increase in patella lead was significantly associated with elevated CHD risk (HR: 1.51, 95%CI: 1.07–2.13); while, the toxic effects of lead tended to decrease among subjects with one or no minor allele. Similarly, we detected a HR of CHD, i.e. 1.62 (95%CI: 1.23–2.14), 1.43 (95%CI: 1.17–1.75), 2.17 (95%CI: 1.51–3.12) and 1.57 (95%CI: 1.27–1.94), respectively, for *HMOX1* rs5995098, *APOE* rs429358, *AGT* rs699 and rs5046, as well as a decreasing trend. In the Benjamini-Hochberg FDR tests, all of the observed significant interactions were substantially reduced and we found borderline significant interactions for *VDR* rs1544410 (*Bsm1*, p = 0.06), rs731236 (*Taq1*, p = 0.06), rs7975232 (*Apa1*, p = 0.06) and *AGT* rs699 (p = 0.06).

For tibia lead, we detected 5 polymorphisms with statistical significance and 2 with borderline significance in additive genetic models (data not shown). Using the dominant genetic model, 4 polymorphisms reached statistical significance and 3 reached borderline significance. The results are similar with regard to *VDR* rs1073581 (*Fok1*), *VDR* rs757343 (*Tru91*), *HMOX1* rs2071749 and *AGT* rs699. We detected 5 different SNPs that significantly altered the relationship between lead exposure and CHD. Among subjects with at least one minor frequency allele, HRs of CHD were 2.04 (95%CI: 1.47, 2.84) for *GSTP1* rs1695, 2.24 (95%CI: 1.45–3.44) for *APOE* rs7412 and 2.81 (95%CI: 1.51, 5.23) for *APOE* rs769446. On the other hand, we found HRs of CHD among subjects with no minor allele for *AGT* rs2493137 (2.06; 95%CI: 1.48–2.87) and *APOE* rs449647 (1.70; 95%CI: 1.36–2.12); whereas, the toxic effects of lead tended to decrease among subjects with one or two minor alleles.

The GRS constructed using significant polymorphisms (GRS 2) ranged from 4 to 17, with a mean of 10. We observed a significant interaction between patella lead levels and GRS 2 (p = <0.0001) (Table 4). For subjects possessing 10 to 12 risk alleles, a HR of CHD was 1.58 (95%CI: 1.19–2.10) times higher with a 2-fold increase in patella bone lead levels; and for subjects possessing 13 to 17 risk alleles, a HR of CHD was 2.77 (95%CI: 1.78, 4.31). However, no significant results were found with people having 4 to 9 risk alleles. Significant effect modification was also detected when we constructed GRS using all the SNPs (GRS 1) (p = 0.006). We also found similar results with tibia lead (S6 Table).

**Table 4:** Adjusted effect estimates^a^ of patella lead, genetic score and interaction between patella lead and genetic risk score.

	N	Patella lead			
Genetic Risk Score 1^b^^,^^d^	393	Hazard ratio	95% CI		P value^e^
**8–16**	113	0.97	0.69	1.36	0.006
**17–19**	143	1.21	0.85	1.72	
**20–27**	137	2.27	1.50	3.42	
**Genetic Risk Score 2**^c^^,^^d^	444				
**4–9**	130	0.82	0.60	1.13	<0.0001
**10–12**	194	1.58	1.19	2.10	
**13–17**	120	2.77	1.78	4.31	

^a^ Adjusted for age, BMI, smoking status (ever/never) and total cholesterol to HDL cholesterol ratio for all models;

^b^ Genetic risk score1 was constructed by using all 22 available SNPs in our study;

^c^ Genetic risk score2 was constructed by using 9 SNPs we found significantly modified the relationship between lead and incident CHD events. The 9 SNPs were *VDR* (rs1544410, rs7975232, rs757343), *HMOX1* (rs2071746, rs2071749, rs5995098), *APOE* (rs429358), and *AGT* (rs699, rs5046).

^d^ Tertiles of genetic risk score with its range in each tertile. Unit for genetic risk score is one risk allele.

^e^ P value for interaction term between genetic risk score and patella lead levels from Wald test in the adjusted Cox proportional hazard models.

In the sensitivity analysis, we did not find significant changes in association between bone lead and incident CHD when incorporating blood pressure into the models. However, this analysis cannot rule out the possibility of blood pressure as an intermediate on the causal pathway from bone lead to CHD events. After replacing the smoking indicator with pack-years, we detected little change in the point estimates. In analyses restricted to participants whose enrollment age was ≤ 45 years along with IPW, we found only slight differences in HRs (data not shown).

Using the observed HRs, we computed post-hoc powers for the gene-lead interaction terms (S7 Table). As expected, our study was underpowered given the sample size of 371 to 545 (power ranged from 0.05 to 0.57). For example, our sample provided 57% power to detect a HR of 2.56 given the allele frequency of 0.55 (*VDR* rs7975232) and the sample size of 520. Because the power calculation was based on unmatched case-control design, actual powers would be higher given our prospective design.

## Discussion

In our study, we systematically analyzed 27 genetic variations in 10 lead-related genes and their modification of the association between cumulative lead and incident CHD. We found significant effect modification by *VDR*, *HMOX1*, *APOE*, *GSTP1* and *AGT* genotype, suggesting that these genes may alter susceptibility to lead-related future development of CHD. We also found significant interactions between GRS and bone lead levels in relation to incident CHD, suggesting that having more risk alleles in lead toxicokinetics and toxicodynamics may enhance cumulative effects of lead on the development of CHD.

This is the first study to construct a GRS using genetic polymorphisms biologically related to lead toxicokinetics and toxicodynamics, and evaluate its interaction with bone lead in relation to incident CHD. Previous studies focused on genetic susceptibility by analyzing SNPs individually. Although the effect of a single locus is found to be relatively small, the combination of relevant SNPs may additively contribute to the lead-related CHD risk. The stronger association found with GRS compared with individual SNPs suggests that GRS has better risk stratification/discrimination power with regards to cardiovascular toxicity of lead. This finding also suggests important clinical and public health implications: genetic profiles can also be considered a useful tool for risk prediction. In addition, lead exposure standards based on average effects in the general population should be revised to protect genetically vulnerable subjects.

Genetic variations related to lead transport might account for the modifications between bone lead levels and CHD risk. Vitamin D receptor (*VDR*) gene is a calcium homeostasis gene and its product serves as a modulator that regulates bone mineral transport [37]. The blood-borne hormonal form of vitamin D, i.e. calcitriol (1α25(OH)2D3), activates VDR. The high affinity VDR interacts with calcitriol to constitute a calcitriol-VDR complex, stimulating the production of calcium-binding proteins such as calbindin-D, which influences calcium transport [56]. During calcium deficiency, calbindin-D is highly expressed. Other divalent cations, especially lead, will bind to the calbindin-D, giving rise to absorption and retention of lead in blood and bone [15]. This supports lead transportation, mediated by *VDR* SNPs, and might explain variation in the lead-CHD association.

Oxidative stress is one of the mechanisms linking genetic variations to lead toxicity. Nitric oxide (NO) plays a central role in regulation of blood pressure by promoting vasodilation and vascular remodeling [57]. Elevation in lead exposure is related to NO inactivation. A mechanistic experiment with two mouse models suggested that *APOE ε4* carriers would have depressed NO bioavailability [43]. In addition, *APOE ε4* might also contribute to lead-related hypertension [58]. Significant results in *APOE* rs429358 are consistent with the underlying theory.

Alleviation of oxidative stress by antioxidants ameliorates the cytotoxicity of ROS and enhances NO bioavailability. It has been recently shown that depletion of glutathione might result in hypertension, with NO inactivation and reduction [59]. Glutathione S-transferase supergene family encodes isoforms modifying lead-induced hypertension, and also contributing to the increased ROS activity and down-regulated NO production in rats [60]. This is also supported by effect modification by *GSTP1* rs1695 in our study.

Heme oxygenase-1 (HMOX1) serves as a protector by reducing free iron levels in cells against oxidative stress. In our study, we found elevated adverse effects of cumulative lead among A-G or A-A genotypes of rs2071749, compared with G-G carriers. However, *HMOX1* length polymorphism was not identified as an effect modifier and interestingly we observed that the cumulative lead effect on CHD risk was increased among subjects with A allele of rs2071746. A single genetic polymorphism may not represent the whole promoter region, unless we incorporate all tagged SNPs, and it is still unknown what function of genetic variations of *HMOX1* promoter might modify associations between lead exposure and CHD risk.

The occurrence of CHD with advancing age, is partially attributed to hypertension caused by the renin-angiotensin system. *AGT* M268T with hypertension was established in Caucasian hypertensive sibling pairs, where *TT* genotype conferred 31% higher risk compared with *MM* genotype [50]. This finding could plausibly be explained by the observation that *TT* genotype associated with higher plasma AGT levels, heightened the production of angiotensin I and II [61], which might also account for our results with regard to the *AGT* gene. Intriguingly, findings from mechanistic and clinical studies showed that calcitriol could serve as a negative endocrine regulator of renin production. Deletion polymorphisms or SNPs in *VDR* gene may affect VDR-vitamin D signaling, which will in turn upregulate activity of RAS. This was supported by augmentation in both renin mRNA and protein levels in the kidney, as well as plasma angiotensin II levels in *VDR* (-/-) mice [62]. Thus RAS over-stimulation mediated by *VDR* polymorphisms may account in part for an association between lead exposure and the development of CHD.

Polymorphisms in *HFE* were not identified as effect modifiers in our study. Although genetic hemochromatosis induced by *HFE* was reported to regulate bone lead absorption, *HFE*-related genetic hemochromatosis would remedy significant bone mineral loss in the elderly groups [63]. Of note, *HFE C282Y* and *H63D* variants were also linked to lower levels of HDL cholesterol, which can stimulate vascular inflammation, through reduction in bone mineral density [64]. It is reasonable to infer that the failure of *HFE* variants to alter lead levels and toxicity results from of perturbation in bone mineral and lipid metabolisms.

*ALAD*, *HMOX1* and *APOE* are hypothesized to alter the circulation of lead, as described above. However, given that bone lead levels were not much different by the genotypes of these genes (S4 Table), it is unlikely that toxicokinetics of bone lead account for the apparent *VDR*, *HMOX1* and *APOE* genotype effects.

Our study has several limitations. In the NAS, all participants were men and predominantly white, so the present findings may not be generalizable to women or other racial/ethnic groups. Moreover, the study sample was reduced somewhat by the requirements of non-missing KXRF bone lead measurements and genotyping. Thus, these prerequisites in our analysis limited the power of our statistical tests. In addition, our findings need to be replicated in other prospective studies with bone lead levels, genotypes and incident CHD data.

## Conclusions

Our results suggest that *VDR*, *HMOX1*, *APOE*, *AGT* and *GSTP1* genes may modify the association between higher bone lead levels and CHD risk. Combined with other evidence that genetic polymorphisms can alter the relationship of CHD-related traits with cumulative exposure to lead, this body of research supports a joint effect of genetic biomarkers of susceptibility and environmental lead exposure on the development of CHD. However, we need to replicate our findings in a separate cohort.

## Supporting Information

S1 File R^2^ of linkage disequilibrium between polymorphisms. (DOC)

S1 Table Predictors included in the inverse probability weighting model. (DOC)

S2 Table Genotype frequencies and incident CHD events by genotype. (DOC)

S3 Table Hardy-Weinberg equilibrium analysis for SNPs. (DOC)

S4 Table Genotype frequencies and bone lead concentrations by genotype. (DOC)

S5 Table Adjusted estimates of CHD per 2-fold increase in patella lead levels, stratified by different gene polymorphisms (no minor allele vs at least one minor allele). (DOC)

S6 Table Adjusted effect estimates of tibia lead, genetic score, and interaction between patella lead and genetic score. (DOC)

S7 Table Statistical powers of the gene-environment interaction terms. (DOC)

## References

[1] Willerson JT, Holmes DR, editors. Coronary Artery Disease [Internet]. London: Springer London; 2015 Available: http://link.springer.com/10.1007/978-1-4471-2828-1

[2] Gunnar F. Nordberg BAF. Nordberg GF, Fowler BA and Nordberg M (eds) Handbook on the Toxicology of Metals 4th ed Elsevier/Academic Press 2014. 2014;

[3] Weisskopf MG, Sparrow D, Hu H, Power MC. Biased Exposure-Health Effect Estimates from Selection in Cohort Studies: Are Environmental Studies at Particular Risk? Environ Health Perspect. 2015; 123: 1113–1122. 10.1289/ehp.1408888 25956004 PMC4629739

[4] Ekong EB, Jaar BG, Weaver VM. Lead-related nephrotoxicity: a review of the epidemiologic evidence. Kidney Int. 2006;70: 2074–2084. 17063179 10.1038/sj.ki.5001809

[5] Rodríguez-Iturbe B, Sindhu RK, Quiroz Y, Vaziri ND. Chronic Exposure to Low Doses of Lead Results in Renal Infiltration of Immune Cells, NF-κB Activation, and Overexpression of Tubulointerstitial Angiotensin II. Antioxid Redox Signal. 2005;7: 1269–1274. 16115032 10.1089/ars.2005.7.1269

[6] Calafat AM. The US National Health and Nutrition Examination Survey and human exposure to environmental chemicals. Int J Hyg Environ Health. 2012;215: 99–101. 10.1016/j.ijheh.2011.08.014 21937270

[7] Menke A, Muntner P, Batuman V, Silbergeld EK, Guallar E. Blood lead below 0.48 micromol/L (10 microg/dL) and mortality among US adults. Circulation. 2006;114: 1388–1394. 16982939 10.1161/CIRCULATIONAHA.106.628321

[8] Program NT, others. NTP Monograph: Health Effects of Low-Level Lead. NTP Monogr. 2012; i. 23964424

[9] Bergdahl IA, Grubb A, Schütz A, Desnick RJ, Wetmur JG, Sassa S, et al Lead binding to delta-aminolevulinic acid dehydratase (ALAD) in human erythrocytes. Pharmacol Toxicol. 1997;81: 153–158. 9353844 10.1111/j.1600-0773.1997.tb02061.x

[10] Feder JN, Penny DM, Irrinki A, Lee VK, Lebrón JA, Watson N, et al The hemochromatosis gene product complexes with the transferrin receptor and lowers its affinity for ligand binding. Proc Natl Acad Sci. 1998;95: 1472–1477. 9465039 10.1073/pnas.95.4.1472 PMC19050

[11] Ashrafunnisa Begum AV. Association of GSTM1 & HMOX-1 Gene Polymorphisms in COPD: A study from South Indian Population. Int J Sci Res. 2014;3: 318–324.

[12] Billionnet C, Sherrill D, Annesi-Maesano I. Estimating the Health Effects of Exposure to Multi-Pollutant Mixture. Ann Epidemiol. 2012;22: 126–141. 10.1016/j.annepidem.2011.11.004 22226033

[13] Park SK, Hu H, Wright RO, Schwartz J, Cheng Y, Sparrow D, et al Iron Metabolism Genes, Low-Level Lead Exposure, and QT Interval. Environ Health Perspect. 2009;117: 80–85. 10.1289/ehp.11559 19165391 PMC2627870

[14] Tanaka G, Aminuddin F, Akhabir L, He J-Q, Shumansky K, Connett JE, et al Effect of heme oxygenase-1 polymorphisms on lung function and gene expression. BMC Med Genet. 2011;12: 117 10.1186/1471-2350-12-117 21902835 PMC3180266

[15] Pounds JG. Effect of lead intoxication on calcium homeostasis and calcium-mediated cell function: a review. Neurotoxicology. 1984;5: 295–331. 6151637

[16] Birdsall RE, Kiley MP, Segu ZM, Palmer CD, Madera M, Gump BB, et al Effects of lead and mercury on the blood proteome of children. J Proteome Res. 2010;9: 4443–4453. 10.1021/pr100204g 20681587 PMC2935177

[17] Jofre-Monseny L, Minihane A-M, Rimbach G. Impact of apoE genotype on oxidative stress, inflammation and disease risk. Mol Nutr Food Res. 2008;52: 131–145. 10.1002/mnfr.200700322 18203129

[18] Stewart WF, Schwartz BS, Simon D, Kelsey K, Todd AC. ApoE genotype, past adult lead exposure, and neurobehavioral function. Environ Health Perspect. 2002;110: 501–505. 10.1289/ehp.02110501 PMC1240838 12003753

[19] Ballatori N. Glutathione mercaptides as transport forms of metals. Adv Pharmacol San Diego Calif. 1994;27: 271–298. 10.1016/s1054-3589(08)61036-4 8068556

[20] Dietze EC, Ibarra C, Dabrowski MJ, Bird A, Atkins WM. Rational Modulation of the Catalytic Activity of A1-1 Glutathione S-Transferase: Evidence for Incorporation of an On-Face (π···HO−Ar) Hydrogen Bond at Tyrosine-9. Biochemistry (Mosc). 1996;35: 11938–11944. 10.1021/bi961073r 8810897

[21] Ercal N, Treeratphan P, Lutz P, Hammond TC, Matthews RH. N-actylcysteine protects Chinese hamster ovary (CHO) cells from lead-induced oxidative stress. Toxicology. 1996;108: 57–64. 8644118 10.1016/s0300-483x(95)03273-i

[22] Sirivarasai J, Wananukul W, Kaojarern S, Chanprasertyothin S, Thongmung N, Ratanachaiwong W, et al Association between Inflammatory Marker, Environmental Lead Exposure, and Glutathione S-Transferase Gene. BioMed Res Int. 2013;2013: e474963. 10.1155/2013/474963 PMC3581115 23484121

[23] Campbell BC, Meredith PA, Scott JJ. Lead exposure and changes in the renin-angiotensin-aldosterone system in man. Toxicol Lett. 1985;25: 25–32. 2986320 10.1016/0378-4274(85)90096-7

[24] Sharifi AM, Darabi R, Akbarloo N, Larijani B, Khoshbaten A. Investigation of circulatory and tissue ACE activity during development of lead-induced hypertension. Toxicol Lett. 2004;153: 233–238. 15451554 10.1016/j.toxlet.2004.04.013

[25] Jhun MA, Hu H, Schwartz J, Weisskopf MG, Nie LH, Sparrow D, et al Effect modification by vitamin D receptor genetic polymorphisms in the association between cumulative lead exposure and pulse pressure: a longitudinal study. Environ Health. 2015;14: 5 10.1186/1476-069X-14-5 25582168 PMC4417283

[26] Kim H-K, Lee H, Kwon J-T, Kim H-J. A polymorphism in AGT and AGTR1 gene is associated with lead-related high blood pressure. J Renin-Angiotensin-Aldosterone Syst JRAAS. 2014. 10.1177/1470320313516174 25031294

[27] Zhang A, Park SK, Wright RO, Weisskopf MG, Mukherjee B, Nie H, et al HFE H63D polymorphism as a modifier of the effect of cumulative lead exposure on pulse pressure: the Normative Aging Study. Environ Health Perspect. 2010;118: 1261–1266. 10.1289/ehp.1002251 20478760 PMC2944087

[28] Hu H, Aro A, Rotnitzky A. Bone lead measured by X-ray fluorescence: epidemiologic methods. Environ Health Perspect. 1995;103: 105–110. 10.1289/ehp.95103s1105 PMC1519344 7621788

[29] Aro AC, Todd AC, Amarasiriwardena C, Hu H. Improvements in the calibration of 109Cd K x-ray fluorescence systems for measuring bone lead in vivo. Phys Med Biol. 1994;39: 2263–2271. 15551552 10.1088/0031-9155/39/12/009

[30] Wilker E, Korrick S, Nie LH, Sparrow D, Vokonas P, Coull B, et al Longitudinal changes in bone lead levels: the VA Normative Aging Study. J Occup Environ Med Am Coll Occup Environ Med. 2011;53: 850–855. 10.1097/JOM.0b013e31822589a9 PMC3159960 21788910

[31] Wetmur JG. Influence of the common human delta-aminolevulinate dehydratase polymorphism on lead body burden. Environ Health Perspect. 1994;102 Suppl 3: 215–219. 7843101 10.1289/ehp.94102s3215 PMC1567421

[32] Alexander BH, Checkoway H, Costa-Mallen P, Faustman EM, Woods JS, Kelsey KT, et al Interaction of blood lead and delta-aminolevulinic acid dehydratase genotype on markers of heme synthesis and sperm production in lead smelter workers. Environ Health Perspect. 1998;106: 213–216. 9495797 10.1289/ehp.98106213 PMC1532970

[33] Schwartz BS, Lee B-K, Stewart W, Ahn K-D, Springer K, Kelsey K. Associations of δ-Aminolevulinic Acid Dehydratase Genotype with Plant, Exposure Duration, and Blood Lead and Zinc Protoporphyrin Levels in Korean Lead Workers. Am J Epidemiol. 1995;142: 738–745. 7572945 10.1093/oxfordjournals.aje.a117705

[34] Hu H, Wu MT, Cheng Y, Sparrow D, Weiss S, Kelsey K. The delta-aminolevulinic acid dehydratase (ALAD) polymorphism and bone and blood lead levels in community-exposed men: the Normative Aging Study. Environ Health Perspect. 2001;109: 827–832. 10.1289/ehp.01109827 PMC1240411 11564619

[35] Pawlas N, Broberg K, Olewińska E, Prokopowicz A, Skerfving S, Pawlas K. Modification by the genes ALAD and VDR of lead-induced cognitive effects in children. NeuroToxicology. 2012;33: 37–43. 10.1016/j.neuro.2011.10.012 22101007

[36] Miyaki K, Lwin H, Masaki K, Song Y, Takahashi Y, Muramatsu M, et al Association between a Polymorphism of Aminolevulinate Dehydrogenase (ALAD) Gene and Blood Lead Levels in Japanese Subjects. Int J Environ Res Public Health. 2009;6: 999–1009. 10.3390/ijerph6030999 19440429 PMC2672380

[37] Hurwitz S. Homeostatic control of plasma calcium concentration. Crit Rev Biochem Mol Biol. 1996;31: 41–100. 8744955 10.3109/10409239609110575

[38] Haynes EN, Kalkwarf HJ, Hornung R, Wenstrup R, Dietrich K, Lanphear BP. Vitamin D receptor Fok1 polymorphism and blood lead concentration in children. Environ Health Perspect. 2003;111: 1665–1669. 14527848 10.1289/ehp.6167 PMC1241691

[39] Cooper GS, Umbach DM. Are vitamin D receptor polymorphisms associated with bone mineral density? A meta-analysis. J Bone Miner Res Off J Am Soc Bone Miner Res. 1996;11: 1841–1849. 10.1002/jbmr.5650111203 8970884

[40] Rezende VB, B F Jr, Montenegro MF, Sandrim VC, Gerlach RF, Tanus-Santos JE. Haplotypes of vitamin D receptor modulate the circulating levels of lead in exposed subjects. Arch Toxicol. 2007;82: 29–36. 17701399 10.1007/s00204-007-0231-4

[41] Waheed A, Parkkila S, Zhou XY, Tomatsu S, Tsuchihashi Z, Feder JN, et al Hereditary hemochromatosis: Effects of C282Y and H63D mutations on association with β2-microglobulin, intracellular processing, and cell surface expression of the HFE protein in COS-7 cells. Proc Natl Acad Sci. 1997;94: 12384–12389. 9356458 10.1073/pnas.94.23.12384 PMC24956

[42] Wright RO, Silverman EK, Schwartz J, Tsaih S-W, Senter J, Sparrow D, et al Association between hemochromatosis genotype and lead exposure among elderly men: the normative aging study. Environ Health Perspect. 2004;112: 746–750. 15121519 10.1289/ehp.6581 PMC1241970

[43] Colton CA, Brown CM, Cook D, Needham LK, Xu Q, Czapiga M, et al APOE and the regulation of microglial nitric oxide production: a link between genetic risk and oxidative stress. Neurobiol Aging. 2002;23: 777–785. 12392781 10.1016/s0197-4580(02)00016-7

[44] Miyata M, Smith JD. Apolipoprotein E allele-specific antioxidant activity and effects on cytotoxicity by oxidative insults and beta-amyloid peptides. Nat Genet. 1996;14: 55–61. 8782820 10.1038/ng0996-55

[45] Tolonen S, Mikkilä V, Laaksonen M, Sievänen H, Mononen N, Hernesniemi J, et al Association of apolipoprotein E promoter polymorphisms with bone structural traits is modified by dietary saturated fat intake—the Cardiovascular Risk in Young Finns study. Bone. 2011;48: 1058–1065. 10.1016/j.bone.2011.01.013 21266206

[46] Theppeang K, Glass TA, Bandeen-Roche K, Todd AC, Rohde CA, Links JM, et al Associations of bone mineral density and lead levels in blood, tibia, and patella in urban-dwelling women. Environ Health Perspect. 2008;116: 784–790. 10.1289/ehp.10977 18560535 PMC2430235

[47] Mannila MN, Mahdessian H, Franco-Cereceda A, Eggertsen G, de Faire U, Syvänen A-C, et al Identification of a functional apolipoprotein E promoter polymorphism regulating plasma apolipoprotein E concentration. Arterioscler Thromb Vasc Biol. 2013;33: 1063–1069. 10.1161/ATVBAHA.112.300353 23430611

[48] Ercal N, Gurer-Orhan H, Aykin-Burns N. Toxic Metals and Oxidative Stress Part I: Mechanisms Involved in Metal-induced Oxidative Damage. Curr Top Med Chem. 2001;1: 529–539. 11895129 10.2174/1568026013394831

[49] Coral-Vázquez RM, Romero Arauz JF, Canizales-Quinteros S, Coronel A, Valencia Villalvazo EY, Hernández Rivera J, et al Analysis of polymorphisms and haplotypes in genes associated with vascular tone, hypertension and oxidative stress in Mexican-Mestizo women with severe preeclampsia. Clin Biochem. 2013;46: 627–632. 10.1016/j.clinbiochem.2012.12.016 23333443

[50] Warrell DA, Cox TM, Firth JD, editors. Oxford Textbook of Medicine [Internet]. Oxford University Press; 2010 Available: http://oxfordmedicine.com/view/10.1093/med/9780199204854.001.1/med-9780199204854

[51] Li H, Du Z, Zhang L, Wu T, Deng Z, Li J, et al The relationship between angiotensinogen gene polymorphisms and essential hypertension in a Northern Han Chinese population. Angiology. 2014;65: 614–619. 10.1177/0003319713491309 23716723

[52] Sheng-jie Nie TW. Haplotype-based case-control study of the human AGTR1 gene and essential hypertension in Han Chinese subjects. Clin Biochem. 2009;43: 253–8. 10.1016/j.clinbiochem.2009.09.027 19833117

[53] Yamada N, Yamaya M, Okinaga S, Nakayama K, Sekizawa K, Shibahara S, et al Microsatellite polymorphism in the heme oxygenase-1 gene promoter is associated with susceptibility to emphysema. Am J Hum Genet. 2000;66: 187–195. 10631150 10.1086/302729 PMC1288325

[54] Schisterman EF, Cole SR, Platt RW. Overadjustment Bias and Unnecessary Adjustment in Epidemiologic Studies. Epidemiol Camb Mass. 2009;20: 488–495. 10.1097/EDE.0b013e3181a819a1 PMC2744485 19525685

[55] Benjamini Y, Hochberg Y. Controlling the False Discovery Rate: A Practical and Powerful Approach to Multiple Testing. J R Stat Soc Ser B Methodol. 1995;57: 289–300.

[56] Wasserman RH, Fullmer CS. Vitamin D and intestinal calcium transport: facts, speculations and hypotheses. J Nutr. 1995;125: 1971S–1979S. 7602379 10.1093/jn/125.suppl_7.1971S

[57] Rudic RD, Shesely EG, Maeda N, Smithies O, Segal SS, Sessa WC. Direct evidence for the importance of endothelium-derived nitric oxide in vascular remodeling. J Clin Invest. 1998;101: 731–736. 9466966 10.1172/JCI1699 PMC508619

[58] Couderc R, Mahieux F, Bailleul S, Fenelon G, Mary R, Fermanian J. Prevalence of apolipoprotein E phenotypes in ischemic cerebrovascular disease. A case-control study. Stroke J Cereb Circ. 1993;24: 661–664. 10.1161/01.str.24.5.661 8488520

[59] Vaziri ND, Wang XQ, Oveisi F, Rad B. Induction of oxidative stress by glutathione depletion causes severe hypertension in normal rats. Hypertension. 2000;36: 142–146. 10904027 10.1161/01.hyp.36.1.142

[60] Hayes JD, Strange RC. Invited Commentary Potential Contribution of the Glutathione S-Transferase Supergene Family to Resistance to Oxidative Stress. Free Radic Res. 1995;22: 193–207. 7757196 10.3109/10715769509147539

[61] Pilbrow AP, Palmer BR, Frampton CM, Yandle TG, Troughton RW, Campbell E, et al Angiotensinogen M235T and T174M gene polymorphisms in combination doubles the risk of mortality in heart failure. Hypertension. 2007;49: 322–327. 17145981 10.1161/01.HYP.0000253061.30170.68

[62] Li YC, Qiao G, Uskokovic M, Xiang W, Zheng W, Kong J. Vitamin D: a negative endocrine regulator of the renin-angiotensin system and blood pressure. J Steroid Biochem Mol Biol. 2004;89–90: 387–392. 15225806 10.1016/j.jsbmb.2004.03.004

[63] Guggenbuhl P, Deugnier Y, Boisdet JF, Rolland Y, Perdriger A, Pawlotsky Y, et al Bone mineral density in men with genetic hemochromatosis and HFE gene mutation. Osteoporos Int. 2005;16: 1809–1814. 15928800 10.1007/s00198-005-1934-0

[64] Choi SH, An JH, Lim S, Koo BK, Park SE, Chang HJ, et al Lower bone mineral density is associated with higher coronary calcification and coronary plaque burdens by multidetector row coronary computed tomography in pre- and postmenopausal women. Clin Endocrinol (Oxf). 2009;71: 644–651. 19226260 10.1111/j.1365-2265.2009.03535.x

