# Supplementary material for: Lead-Related Genetic Loci, Cumulative Lead Exposure and Incident Coronary Heart Disease: The Normative Aging Study
Source: PLoS One. 2016 Sep 1;11(9):e0161472. doi: 10.1371/journal.pone.0161472 (PMC5008632; doi:10.1371/journal.pone.0161472)
Supplement: S1 File — (DOC) [file pone.0161472.s001.doc]

**Table A. R^2^ of linkage disequilibrium between polymorphisms of the *VDR*** gene

| **R^2^** | **rs731236** | **rs7975232** | **rs1073581** | **rs757343** |
| --- | --- | --- | --- | --- |
| **rs1544410** | 0.97 | 0.75 | 0.02 | 0.02 |
| **rs731236** |  | 0.75 | 0.04 | 0.03 |
| **rs7975232** |  |  | 0.001 | 0.01 |
| **rs1073581** |  |  |  | 0.99 |

**Table B.** **R^2^ of linkage disequilibrium between polymorphisms of the *HMOX1* gene**

| **R^2^** | **rs2071747** | **rs2071749** | **rs5995098** |
| --- | --- | --- | --- |
| **rs2071746** | 0.22 | 0.79 | 0.73 |
| **rs2071747** |  | 0.18 | 0.14 |
| **rs2071749** |  |  | 0.59 |

**Table C.** **R^2^ of linkage disequilibrium between polymorphisms of the *APOE* gene**

| **R^2^** | **rs405509** | **rs449647** | **rs7412** | **rs429358** | **rs769446** |
| --- | --- | --- | --- | --- | --- |
| **rs440446** | 0.79 | 0.15 | 0.21 | 0.21 | 0.03 |
| **rs405509** |  | 0.05 | 0.27 | 0.15 | 0.04 |
| **rs449647** |  |  | 0.20 | 0.14 | 0.09 |
| **rs7412** |  |  |  | 0.08 | 0.22 |
| **rs429358** |  |  |  |  | 0.04 |

**Table D.** **R^2^ of linkage disequilibrium between polymorphisms of the *AGT* gene**

| **R^2^** | **rs5046** | **rs5050** | **rs2493137** |
| --- | --- | --- | --- |
| **rs699** | 0.49 | 0.43 | 0.74 |
| **rs5046** |  | 0.10 | 0.65 |
| **rs5050** |  |  | 0.23 |
