# Supplementary material for: Lead-Related Genetic Loci, Cumulative Lead Exposure and Incident Coronary Heart Disease: The Normative Aging Study
Source: PLoS One. 2016 Sep 1;11(9):e0161472. doi: 10.1371/journal.pone.0161472 (PMC5008632; doi:10.1371/journal.pone.0161472)
Supplement: S1 Table — (DOC) [file pone.0161472.s002.doc]

**S1 Table. Predictors included in the inverse probability weighting model [1].**

| **Predictors** |
| --- |
| Age and its square |
| Number of NAS visit |
| Year of NAS visit, its square and its cube |
| Year of first diagnosis of diabetes |
| Indicator of first CHD event |
| White collar job |
| Marital status |
| Abnormal fasting glucose |
| Abnormal hematocrit |
| Blood protein |
| Serum calcium |
| Body mass index |
| Abnormal waist circumference |

# Reference

1. Weisskopf MG, Sparrow D, Hu H, Power MC. Biased Exposure-Health Effect Estimates from Selection in Cohort Studies: Are Environmental Studies at Particular Risk? Environ Health Perspect. 2015;123: 1113–1122.
