# Supplementary material for: Lead-Related Genetic Loci, Cumulative Lead Exposure and Incident Coronary Heart Disease: The Normative Aging Study
Source: PLoS One. 2016 Sep 1;11(9):e0161472. doi: 10.1371/journal.pone.0161472 (PMC5008632; doi:10.1371/journal.pone.0161472)
Supplement: S2 Table — (DOC) [file pone.0161472.s003.doc]

**S2 Table.** **Genotype frequencies and incident CHD events by genotype.**

| Minor Allele Frequency | | | | | | | | | |
| --- | --- | --- | --- | --- | --- | --- | --- | --- | --- |
| **SNP** | **Minor Frequency Allele** | **Gene Symbol** | **SNP location** | **No CHD** | | | **CHD** | | |
|  |  |  |  | **No Allele** | **One Allele** | **Two alleles** | **No Allele** | **One Allele** | **Two alleles** |
|  |  |  |  | **N (%)** | | | **N (%)** | | |
| rs1800435 | C | *ALAD* | Exon 4^a^ | 344 (82.1) | 70 (16.7) | 5 (1.2) | 118 (90.1) | 11 (8.4) | 2 (1.5) |
| rs1544410 | A | *VDR* | Intron 8 | 125 (33.2) | 176 (46.8) | 75 (20.0) | 49 (39.8) | 59 (48.0) | 15 (12.2) |
| rs731236 | C | *VDR* | Exon 9^a^ | 128 (32.4) | 199 (50.4) | 68 (17.2) | 50 (38.2) | 64 (48.8) | 17 (13.0) |
| rs7975232 | C | *VDR* | Intron 8 | 111 (28.2) | 211 (53.5) | 72 (18.3) | 42 (32.1) | 57 (43.5) | 32 (24.4) |
| rs1073581 | A | *VDR* | Exon 2^a^ | 142 (36.6) | 194 (50.0) | 52 (13.4) | 48 (38.1) | 66 (52.4) | 12 (9.5) |
| rs757343 | A | *VDR* | Intron 8 | 147 (37.7) | 191 (49.0) | 52 (13.3) | 50 (38.2) | 69 (52.7) | 12 (9.1) |
| rs1799945 | G | *HFE* | Exon 2^a^ | 310 (79.3) | 71 (18.2) | 10 (2.5) | 91 (74.0) | 29 (23.6) | 3 (2.4) |
| rs1800562 | A | *HFE* | Exon 4^a^ | 340 (86.7) | 47 (12.0) | 5 (1.3) | 108 (87.8) | 15 (12.2) | 0 (0.0) |
| rs2071746 | T | *HMOX1* | Promoter | 117 (29.8) | 192 (49.0) | 83 (21.2) | 40 (31.0) | 59 (45.7) | 30 (23.3) |
| rs2071747 | C | *HMOX1* | Exon 1^a^ | 358 (92.5) | 29 (7.5) | 0 (0.0) | 115 (89.8) | 13 (10.2) | 0 (0.0) |
| rs2071749 | A | *HMOX1* | Intron 3 | 129 (33.1) | 185 (47.4) | 76 (19.5) | 46 (35.7) | 56 (43.4) | 27 (20.9) |
| rs5995098 | G | *HMOX1* | Intron 4 | 188 (47.7) | 159 (40.4) | 47 (11.9) | 60 (46.2) | 58 (44.6) | 12 (9.2) |
| rs440446 | C | *APOE* | Intron 1 | 155 (38.8) | 181 (45.2) | 64 (16.0) | 44 (34.9) | 62 (49.2) | 20 (15.9) |
| rs405509 | C | *APOE* | Promoter | 104 (25.4) | 206 (50.2) | 100 (24.4) | 29 )22.5) | 69 (53.5) | 31 (24.0) |
| rs449647 | A | *APOE* | Promoter | 237 (61.1) | 141 (36.3) | 10 (2.6) | 72 (56.2) | 45 (35.2) | 11 (8.6) |
| rs7412 | T | *APOE* | Exon 4^a^ | 357 (86.2) | 57 (13.8) | 0 (0.0) | 105 (80.2) | 26 (19.8) | 0 (0.0) |
| rs429358 | C | *APOE* | Exon 4^a^ | 293 (77.1) | 84 (22.1) | 3 (0.8) | 95 (76.6) | 26 (21.0) | 3 (2.4) |
| rs769446 | C | *APOE* | Promoter | 334 (85.9) | 50 (12.8) | 5 (1.3) | 108 (86.4) | 16 (12.8) | 1 (0.8) |
| rs1695 | G | *GSTP1* | Exon 5^a^ | 179 (46.9) | 158 (41.3) | 45 (11.8) | 60 (50.4) | 52 (43.7) | 7 (5.9) |
| rs699 | C | *AGT* | Exon 2^a^ | 117 (31.7) | 172 (46.6) | 80 (21.7) | 37 (30.6) | 59 (48.8) | 25 (20.6) |
| rs5046 | T | *AGT* | Promoter | 282 (76.4) | 84 (22.8) | 3 (0.8) | 93 (75.6) | 28 (22.8) | 2 (1.6) |
| rs5050 | G | *AGT* | Promoter | 248 (67.8) | 114 (31.1) | 4 (1.1) | 84 (68.9) | 38 (31.1) | 0 (0.0) |
| rs2493137 | C | *AGT* | Promoter | 165 (44.7) | 166 (45.0) | 38 (10.3) | 55 (45.5) | 56 (46.3) | 10 (8.2) |

^a^ Amino acid substitution in exons: rs1800435 Lys59Asn, rs731236 Ile352Ile, rs1799945 His63Asp, rs1800562 Cys282Try, rs2071747 Asp7His, rs7412 Arg176Cys, rs429358 Cys130Arg, rs1695 Ile105Val, and rs699 Met268Thr^b^.

^b^ In most literature, the SNP location is M235T. However, based on numbering in current databases, it changes into amino acid 268.
