# Supplementary material for: Lead-Related Genetic Loci, Cumulative Lead Exposure and Incident Coronary Heart Disease: The Normative Aging Study
Source: PLoS One. 2016 Sep 1;11(9):e0161472. doi: 10.1371/journal.pone.0161472 (PMC5008632; doi:10.1371/journal.pone.0161472)
Supplement: S3 Table — (DOC) [file pone.0161472.s004.doc]

**S3 Table. Hardy-Weinberg equilibrium analysis for SNPs**

| **SNP** | **P value^a^** |
| --- | --- |
| **rs1800435** | 0.12 |
| **rs1544410** | 0.49 |
| **rs731236** | 0.46 |
| **rs7975232** | 0.49 |
| **rs1073581** | 0.08 |
| **rs757343** | 0.12 |
| **rs1799945** | 0.03 |
| **rs1800562** | 0.09 |
| **rs2071746** | 0.50 |
| **rs2071747** | 0.33 |
| **rs2071749** | 0.23 |
| **rs5995098** | 0.27 |
| **rs440446** | 0.50 |
| **rs405509** | 0.64 |
| **rs449647** | 0.28 |
| **rs7412** | 0.05 |
| **rs429358** | 0.56 |
| **rs769446** | 0.06 |
| **rs1695** | 0.56 |
| **rs699** | 0.29 |
| **rs5046** | 0.003 |
| **rs5050** | 0.29 |
| **rs2493137** | 0.46 |
| **rs12695908** | 0.69 |

^a^ P value from Chi-square test
