# Supplementary material for: Lead-Related Genetic Loci, Cumulative Lead Exposure and Incident Coronary Heart Disease: The Normative Aging Study
Source: PLoS One. 2016 Sep 1;11(9):e0161472. doi: 10.1371/journal.pone.0161472 (PMC5008632; doi:10.1371/journal.pone.0161472)
Supplement: S4 Table — (DOC) [file pone.0161472.s005.doc]

**S4 Table.** Genotype frequencies and bone lead concentrations by genotype

|  |  | **Minor Allele Frequency** | | | | | |  |  | | | | | |  |
| --- | --- | --- | --- | --- | --- | --- | --- | --- | --- | --- | --- | --- | --- | --- | --- |
|  |  | **Patella lead** | | | | | |  | **Tibia lead** | | | | | |  |
| **SNP** | **Gene Symbol** | **No Allele** | | **One Allele** | | **Two Alleles** | | **P^a^** | **No Allele** | | **One Allele** | | **Two Alleles** | | **P^a^** |
|  |  | N | Mean±SD^b^ | N | Mean±SD | N | Mean±SD |  | N | Mean±SD | N | Mean±SD | N | Mean±SD |  |
| **rs1800435** | *ALAD* | 457 | 29.9±18.9 | 81 | 26.4±15.1 | 7 | 33.9±15.1 | 0.23 | 461 | 20.9±12.8 | 81 | 18.6±9.6 | 7 | 25.3±8.8 | 0.18 |
| **rs1544410** | *VDR* | 172 | 30.7±21.8 | 234 | 28.5±16.0 | 88 | 30.5±19.3 | 0.45 | 174 | 21.4±13.0 | 234 | 20.1±11.9 | 90 | 21.1±13.7 | 0.56 |
| **rs731236** | *VDR* | 176 | 31.1±21.7 | 262 | 28.1±15.5 | 83 | 30.3±19.7 | 0.23 | 178 | 21.4±12.8 | 262 | 20.0±11.7 | 85 | 20.5±14.1 | 0.53 |
| **rs7975232** | *VDR* | 151 | 30.0±19.4 | 265 | 27.9±16.0 | 104 | 33.0±22.5 | 0.06 | 153 | 20.9±14.0 | 267 | 19.6±11.0 | 104 | 22.7±13.3 | 0.10 |
| **rs1073581** | *VDR* | 189 | 28.1±18.2 | 256 | 30.3±17.8 | 64 | 27.2±17.6 | 0.30 | 190 | 19.6±12.6 | 259 | 21.1±12.1 | 64 | 19.8±11.7 | 0.38 |
| **rs757343** | *VDR* | 196 | 29.0±18.5 | 256 | 30.0±17.4 | 64 | 27.4±17.6 | 0.56 | 197 | 20.1±12.6 | 259 | 20.9±11.9 | 64 | 20.0±11.6 | 0.73 |
| **rs1799945** | *HFE* | 399 | 30.1±18.3 | 97 | 26.9±17.5 | 13 | 27.4±11.7 | 0.26 | 400 | 20.8±12.4 | 100 | 19.7±11.8 | 13 | 17.6±7.1 | 0.48 |
| **rs1800562** | *HFE* | 443 | 29.7±18.3 | 62 | 26.5±14.7 | 5 | 36.0±22.5 | 0.31 | 448 | 20.6±12.5 | 61 | 19.5±9.9 | 5 | 24.8±10.7 | 0.59 |
| **rs2071746** | *HMOX1* | 157 | 29.3±15.8 | 248 | 28.6±17.8 | 111 | 31.7±22.9 | 0.35 | 157 | 20.4±12.5 | 250 | 20.1±11.6 | 113 | 22.1±14.3 | 0.37 |
| **rs2071747** | *HMOX1* | 468 | 29.3±17.8 | 42 | 28.2±18.5 | 0 | 0.0±0.0 | 0.71 | 472 | 20.5±12.2 | 42 | 19.1±11.1 | 0 | 0.0±0.0 | 0.47 |
| **rs2071749** | *HMOX1* | 173 | 31.0±20.8 | 238 | 28.4±18.1 | 103 | 29.3±15.9 | 0.38 | 175 | 21.3±12.9 | 240 | 19.9±11.9 | 103 | 20.5±13.1 | 0.52 |
| **rs5995098** | *HMOX1* | 248 | 28.0±15.6 | 212 | 30.3±19.5 | 59 | 31.3±20.4 | 0.26 | 248 | 19.5±11.6 | 216 | 21.5±12.6 | 59 | 20.9±12.5 | 0.20 |
| **rs440446** | *APOE* | 196 | 28.9±17.2 | 243 | 29.5±17.4 | 82 | 28.8±17.8 | 0.91 | 199 | 19.9±12.7 | 242 | 20.7±10.9 | 84 | 21.1±13.1 | 0.67 |
| **rs405509** | *APOE* | 132 | 28.9±18.8 | 273 | 30.2±17.3 | 129 | 27.5±17.1 | 0.35 | 133 | 19.8±13.9 | 274 | 21.0±11.4 | 131 | 20.3±11.4 | 0.64 |
| **rs449647** | *APOE* | 307 | 30.1±19.5 | 186 | 28.7±17.3 | 20 | 27.1±13.8 | 0.58 | 30.9 | 21.0±13.3 | 185 | 20.4±11.5 | 21 | 16.3±9.7 | 0.25 |
| **rs7412** | *APOE* | 457 | 29.7±18.2 | 83 | 27.1±15.1 | 0 | 0.0±0.0 | 0.27 | 461 | 20.6±12.3 | 83 | 20.2±10.9 | 0 | 0.0±0.0 | 0.68 |
| **rs429358** | *APOE* | 385 | 29.4±18.6 | 109 | 29.7±19.3 | 6 | 30.0±22.8 | 0.98 | 387 | 20.3±12.6 | 110 | 21.4±12.8 | 6 | 19.3±9.5 | 0.71 |
| **rs769446** | *APOE* | 437 | 29.9±18.3 | 66 | 27.3±16.0 | 6 | 19.2±17.4 | 0.21 | 441 | 20.6±12.4 | 66 | 21.0±10.3 | 6 | 11.3±13.7 | 0.17 |
| **rs1695** | *GSTP1* | 236 | 28.7±17.5 | 209 | 30.2±18.5 | 51 | 26.5±14.0 | 0.36 | 238 | 19.8±11.4 | 210 | 21.6±13.2 | 52 | 18.7±10.4 | 0.17 |
| **rs699** | *AGT* | 152 | 29.3±16.4 | 229 | 29.8±19.5 | 104 | 27.6±16.5 | 0.59 | 154 | 20.6±11.4 | 230 | 20.4±12.8 | 105 | 20.4±12.3 | 0.99 |
| **rs5046** | *AGT* | 371 | 29.5±17.9 | 111 | 27.9±17.9 | 5 | 22.8±8.6 | 0.51 | 375 | 21.1±12.4 | 112 | 18.6±11.4 | 5 | 19.6±9.9 | 0.17 |
| **rs5050** | *AGT* | 328 | 29.7±18.8 | 151 | 28.5±16.0 | 4 | 16.5±7.7 | 0.28 | 332 | 20.7±12.6 | 151 | 19.9±11.3 | 4 | 20.5±7.0 | 0.79 |
| **rs2493137** | *AGT* | 217 | 28.9±16.5 | 221 | 29.6±19.3 | 47 | 29.0±18.2 | 0.90 | 220 | 20.0±11.3 | 221 | 20.6±12.8 | 48 | 22.6±14.2 | 0.43 |

^a^ P values derived from ANOVA

^b^ SD: standard deviation; unit for mean and SD is μg/g.
