# Supplementary material for: Lead-Related Genetic Loci, Cumulative Lead Exposure and Incident Coronary Heart Disease: The Normative Aging Study
Source: PLoS One. 2016 Sep 1;11(9):e0161472. doi: 10.1371/journal.pone.0161472 (PMC5008632; doi:10.1371/journal.pone.0161472)
Supplement: S5 Table — (DOC) [file pone.0161472.s006.doc]

**S5 Table.** **Adjusted estimates^a^ of CHD per 2-fold increase in patella lead levels, stratified by different gene polymorphisms (no minor allele vs at least one minor allele).**

| **Associations by genotype of each lead-related SNPs** |  |  |  | |  |
| --- | --- | --- | --- | --- | --- |
| **Genotype** | **N** | **Hazard Ratio^b^** | **95% Confidence Interval** | | **P value^c^** |
| ***Vitamin D (1,25-dihydroxyvitamin D3) receptor gene (VDR gene)*** |  |  |  | |  |
| ***VDR rs1544410 (Bsm1)*** | 494 |  |  |  | 0.004 |
| **No minor allele** |  | 0.97 | 0.73 | 1.28 |  |
| **At least one minor allele** |  | 1.65 | 1.31 | 2.08 |  |
| ***VDR rs731236 (Taq1)*** | 521 |  |  |  | 0.008 |
| **No minor allele** |  | 1.00 | 0.76 | 1.32 |  |
| **At least one minor allele** |  | 1.61 | 1.29 | 2.02 |  |
| ***VDR rs7975232 (Apa1)*** | 520 |  |  |  | 0.41 |
| **No minor allele** |  | 1.48 | 1.11 | 1.96 |  |
| **At least one minor allele** |  | 1.28 | 1.04 | 1.57 |  |
| ***VDR rs1073581 (Fok1)*** | 509 |  |  |  | 0.25 |
| **No minor allele** |  | 1.20 | 0.91 | 1.57 |  |
| **At least one minor allele** |  | 1.47 | 1.17 | 1.83 |  |
| ***VDR rs757343 (Tru91)*** | 516 |  |  |  | 0.18 |
| **No minor allele** |  | 1.17 | 0.90 | 1.53 |  |
| **At least one minor allele** |  | 1.48 | 1.18 | 1.85 |  |
| ***δ-aminolevulinic acid dehydratase gene (ALAD gene)*** |  |  |  |  |  |
| ***ALAD rs1833435*** | 545 |  |  |  | 0.95 |
| **No minor allele** |  | 1.34 | 1.12 | 1.60 |  |
| **At least one minor allele** |  | 1.31 | 0.75 | 2.29 |  |
| ***Hemochromatosis gene (HFE gene)*** |  |  |  |  |  |
| ***HFE rs1799945 (H63D)*** | 509 |  |  |  | 0.23 |
| **No minor allele** |  | 1.41 | 1.15 | 1.73 |  |
| **At least one minor allele** |  | 1.11 | 0.79 | 1.55 |  |
| ***HFE rs1800562 (C282Y)*** | 510 |  |  |  | 0.23 |
| **No minor allele** |  | 1.36 | 1.13 | 1.64 |  |
| **At least one minor allele** |  | 0.97 | 0.57 | 1.65 |  |
| ***Heme oxygenase 1 gene (HMOX1 gene)*** |  |  |  |  |  |
| ***HMOX1 rs2071746*** | 516 |  |  |  | 0.39 |
| **No minor allele** |  | 1.51 | 1.07 | 2.14 |  |
| **At least one minor allele** |  | 1.28 | 1.05 | 1.55 |  |
| ***HMOX1 rs2071749*** | 514 |  |  |  | 0.02 |
| **No minor allele** |  | 1.02 | 0.78 | 1.35 |  |
| **At least one minor allele** |  | 1.51 | 1.22 | 1.86 |  |
| ***HMOX1 rs5995098*** | 519 |  |  |  | 0.07 |
| **No minor allele** |  | 1.63 | 1.23 | 2.14 |  |
| **At least one minor allele** |  | 1.18 | 0.95 | 1.47 |  |
| ***HMOX1 rs2071747*** | 510 |  |  |  | 0.82 |
| **No minor allele** |  | 1.36 | 1.13 | 1.63 |  |
| **At least one minor allele** |  | 1.45 | 0.81 | 2.61 |  |
| ***HMOX1 length polymorphisms*** | 541 |  |  |  | 0.18 |
| **S or M alleles** |  | 1.46 | 1.20 | 1.77 |  |
| **Any L allele** |  | 1.09 | 0.74 | 1.60 |  |
| ***Alipoprotein E gene (APOE gene)*** |  |  |  |  |  |
| ***APOE rs429358*** | 500 |  |  |  | 0.09 |
| **No minor allele** |  | 1.43 | 1.17 | 1.76 |  |
| **At least one minor allele** |  | 1.05 | 0.77 | 1.43 |  |
| ***APOE rs440446*** | 521 |  |  |  | 0.52 |
| **No minor allele** |  | 1.25 | 0.95 | 1.63 |  |
| **At least one minor allele** |  | 1.40 | 1.11 | 1.77 |  |
| ***APOE rs405509*** | 534 |  |  |  | 0.63 |
| **No minor allele** |  | 1.22 | 0.86 | 1.74 |  |
| **At least one minor allele** |  | 1.35 | 1.10 | 1.65 |  |
| ***APOE rs449647*** | 513 |  |  |  | 0.43 |
| **No minor allele** |  | 1.29 | 1.05 | 1.60 |  |
| **At least one minor allele** |  | 1.50 | 1.11 | 2.01 |  |
| ***APOE rs7412*** | 540 |  |  |  | 0.53 |
| **No minor allele** |  | 1.34 | 1.10 | 1.64 |  |
| **At least one minor allele** |  | 1.53 | 1.07 | 2.19 |  |
| ***APOE rs769446*** | 509 |  |  |  | 0.94 |
| **No minor allele** |  | 1.32 | 1.08 | 1.61 |  |
| **At least one minor allele** |  | 1.34 | 0.94 | 1.92 |  |
| ***Angiotensinogen gene (AGT gene)*** |  |  |  |  |  |
| ***AGT rs699*** | 485 |  |  |  | 0.01 |
| **No minor allele** |  | 2.17 | 1.50 | 3.12 |  |
| **At least one minor allele** |  | 1.26 | 1.03 | 1.54 |  |
| ***AGT rs5046*** | 487 |  |  |  | 0.06 |
| **No minor allele** |  | 1.56 | 1.27 | 1.94 |  |
| **At least one minor allele** |  | 1.12 | 0.84 | 1.51 |  |
| ***AGT rs5050*** | 483 |  |  |  | 0.84 |
| **No minor allele** |  | 1.36 | 1.09 | 1.69 |  |
| **At least one minor allele** |  | 1.41 | 1.03 | 1.94 |  |
| ***AGT rs2493137*** | 485 |  |  |  | 0.31 |
| **No minor allele** |  | 1.61 | 1.20 | 2.15 |  |
| **At least one minor allele** |  | 1.33 | 1.06 | 1.67 |  |
| ***Angiotensin II receptor 1 gene (AGTR1 gene)*** |  |  |  |  |  |
| ***AGTR1 rs12695908*** | 486 |  |  |  | 0.72 |
| **No minor allele** |  | 1.43 | 1.20 | 1.74 |  |
| **At least one minor allele** |  | 1.27 | 0.63 | 2.55 |  |
| ***Glutathione S-transferase pi 1 gene (GSTP1 gene)*** |  |  |  |  |  |
| ***GSTP1 rs1695*** | 496 |  |  |  | 0.40 |
| **No minor allele** |  | 1.39 | 1.10 | 1.76 |  |
| **At least one minor allele** |  | 1.19 | 0.91 | 1.57 |  |
| ***Glutathione S-Transferase Theta 1 (GSTT1 gene)*** |  |  |  |  |  |
| ***GSTT1*** | 386 |  |  |  | 0.16 |
| **Deletion** |  | 1.21 | 0.84 | 1.76 |  |
| **No deletion** |  | 1.65 | 1.32 | 2.08 |  |
| ***Glutathione S-Transferase Mu 1 (GSTM1 gene)*** |  |  |  |  |  |
| ***GSTM1*** | 519 |  |  |  | 0.15 |
| **Deletion** |  | 1.56 | 1.21 | 2.01 |  |
| **No deletion** |  | 1.21 | 0.95 | 1.54 |  |

^a^ Hazard ratio and 95% confidence interval with adjustment for age, BMI, smoking status (ever/never) and total cholesterol to HDL cholesterol ratio for all models.

^b^ Hazard ratio indicates HR of CHD events for 2-fold increase in lead levels.

^c^ P value from Wald test in the adjusted Cox proportional hazard models, when genotype indicators treated as a binary variable.
