# Supplementary material for: Lead-Related Genetic Loci, Cumulative Lead Exposure and Incident Coronary Heart Disease: The Normative Aging Study
Source: PLoS One. 2016 Sep 1;11(9):e0161472. doi: 10.1371/journal.pone.0161472 (PMC5008632; doi:10.1371/journal.pone.0161472)
Supplement: S6 Table — (DOC) [file pone.0161472.s007.doc]

**S6 Table. Adjusted^a^ hazard ratios of incident CHD per 2-fold increase in tibia lead by tertiles of genetic risk score.**

|  | **N** | **Tibia Lead** |  | |  |
| --- | --- | --- | --- | --- | --- |
| **Genetic Risk Score1^b,d^** | 394 | **Hazard ratio** | **95% Confidence Interval** | | **P value^e^** |
| 10 -18 | 142 | 0.81 | 0.61 | 1.08 | <0.0001 |
| 19 – 20 | 97 | 3.31 | 2.14 | 5.12 |  |
| 21 - 27 | 155 | 2.32 | 1.46 | 3.68 |  |
|  |  |  |  |  |  |
| **Genetic Risk Score2^c,d^** | 447 |  |  |  |  |
| 1 – 5 | 139 | 1.08 | 0.79 | 1.47 | 0.0029 |
| 6 - 7 | 138 | 2.07 | 1.48 | 2.89 |  |
| 8 - 11 | 101 | 2.41 | 1.53 | 3.78 |  |

^a^ Adjusted for age, BMI and total cholesterol to HDL cholesterol ratio for all models;

^b^ Genetic risk score1 was constructed by using all 22 available SNPs in our study;

^c^ Genetic risk score2 was constructed by using 8 SNPs we found significantly modified the relationship between lead and incident CHD events. The 8 SNPs were *VDR* (rs757343), *HMOX1* (rs2071749), *APOE* (rs7412, rs449647, rs769446), *GSTP1* (rs1695), and *AGT* (rs699, rs2493137).

^d^ Tertiles of genetic risk score with its range in each tertile. Unit for genetic risk score is one risk allele.

^e^ P value for interaction term between genetic risk score and patella lead levels from Wald test in the adjusted Cox proportional hazard models.
