# Supplementary material for: Lead-Related Genetic Loci, Cumulative Lead Exposure and Incident Coronary Heart Disease: The Normative Aging Study
Source: PLoS One. 2016 Sep 1;11(9):e0161472. doi: 10.1371/journal.pone.0161472 (PMC5008632; doi:10.1371/journal.pone.0161472)
Supplement: S7 Table — (DOC) [file pone.0161472.s008.doc]

**S7 Table. Statistical powers^a^ of the gene-environment interaction terms^b^.**

| **Associations by genotype of each lead-related SNPs** | **No. of participants^c^** | **No. of cases^c^** | **Risk allele frequency** | **Observed hazard ratio for interaction** | **Post-hoc power for interaction** |
| --- | --- | --- | --- | --- | --- |
| ***Vitamin D (1,25-dihydroxyvitamin D3) receptor gene (VDR gene)*** |  |  |  |  |  |
| ***VDR rs1544410 (Bsm1)*** | 494 | 122 | 0.41 | 2.16 | 0.46 |
| ***VDR rs731236 (Taq1)*** | 521 | 130 | 0.41 | 1.99 | 0.43 |
| ***VDR rs7975232 (Apa1)*** | 520 | 130 | 0.55 | 2.56 | 0.57 |
| ***VDR rs1073581 (Fok1)*** | 509 | 125 | 0.38 | 1.34 | 0.13 |
| ***VDR rs757343 (Tru91)*** | 516 | 130 | 0.37 | 1.40 | 0.16 |
| ***δ-aminolevulinic acid dehydratase gene (ALAD gene)*** |  |  |  |  |  |
| ***ALAD rs1833435*** | 545 | 130 | 0.09 | 0.98 | 0.05 |
| ***Hemochromatosis gene (HFE gene)*** |  |  |  |  |  |
| ***HFE rs1799945(H63D)*** | 509 | 122 | 0.12 | 0.70 | 0.13 |
| ***HFE rs1800562 (C282Y)*** | 510 | 122 | 0.07 | 0.61 | 0.14 |
| ***Heme oxygenase 1 gene (HMOX1 gene)*** |  |  |  |  |  |
| ***HMOX1 rs2071746*** | 516 | 128 | 0.46 | 0.78 | 0.10 |
| ***HMOX1 rs2071749*** | 514 | 128 | 0.43 | 1.75 | 0.32 |
| ***HMOX1 rs5995098*** | 519 | 129 | 0.68 | 0.97 | 0.05 |
| ***HMOX1 rs2071747*** | 510 | 127 | 0.04 | 1.11 | 0.06 |
| ***Alipoprotein E gene (APOE gene)*** |  |  |  |  |  |
| ***APOE rs429358*** | 500 | 123 | 0.88 | 1.78 | 0.09 |
| ***APOE rs440446*** | 521 | 125 | 0.39 | 1.18 | 0.08 |
| ***APOE rs405509*** | 534 | 128 | 0.50 | 1.15 | 0.06 |
| ***APOE rs449647*** | 513 | 127 | 0.22 | 1.23 | 0.09 |
| ***APOE rs7412*** | 540 | 130 | 0.08 | 1.21 | 0.07 |
| ***APOE rs769446*** | 509 | 124 | 0.08 | 1.02 | 0.05 |
| ***Angiotensinogen gene (AGT gene)*** |  |  |  |  |  |
| ***AGT rs699*** | 485 | 120 | 0.55 | 1.43 | 0.14 |
| ***AGT rs5046*** | 487 | 122 | 0.88 | 1.57 | 0.07 |
| ***AGT rs5050*** | 485 | 121 | 0.16 | 1.06 | 0.05 |
| ***AGT rs2493137*** | 486 | 120 | 0.68 | 1.57 | 0.14 |
| ***Angiotensin II receptor 1 gene (AGTR1 gene)*** |  |  |  |  |  |
| ***AGTR1 rs12695908*** | 486 | 121 | 0.95 | 1.13 | 0.05 |
| ***Glutathione S-transferase pi 1 gene (GSTP1 gene)*** |  |  |  |  |  |
| ***GSTP1 rs1695*** | 496 | 118 | 0.31 | 0.80 | 0.09 |
| **Genetic risk score^d^ (GRS)** |  |  |  |  |  |
| **GRS 1** | 371 | 88 | 0.54 | 2.05 | 0.28 |
| **GRS 2** | 418 | 101 | 0.49 | 3.28 | 0.55 |

^a^ The statistical powers were calculated using Quanto 1.2.4 under the gene-environment interaction hypothesis with a two-sided type-1 error of 0.05. Since the Quanto 1.2.4 can only capture case-control study designs, we assumed our study design was a standard unmatched case-control with a dominant inheritance model (i.e. risk allele carriers vs. participants with no risk allele).

^b^ Length polymorphisms and deletion polymorphisms were excluded, since the Quanto 1.2.4 could not compute their statistical powers.

^c^ The numbers of cases and participants varied due to missing values.

^d^ Genetic risk score was categorized by tertiles. Subjects with GRS1 equal to 8-16 approximate participants with no risk allele; subjects with GRS1 equal to 17-19 approximate participants carrying one risk allele; subjects with GRS1 equal to 20-27 approximate participants carrying two risk alleles. Similarly, subjects with GRS2 equal to 4-9 approximate participants with no risk allele; subjects with GRS2 equal to 10-12 approximate participants carrying one risk allele; subjects with GRS2 equal to 13-17 approximate participants carrying two risk alleles.
